# Looking for a safe haven: tail-anchored proteins and their membrane insertion pathways

**DOI:** 10.1093/plphys/kiab298

**Published:** 2021-06-21

**Authors:** Dietmar G Mehlhorn, Lisa Y Asseck, Christopher Grefen

**Affiliations:** Faculty of Biology and Biotechnology, Molecular and Cellular Botany, University of Bochum, Universitätsstraße 150, 44780 Bochum, Germany

## Abstract

Insertion of membrane proteins into the lipid bilayer is a crucial step during their biosynthesis. Eukaryotic cells face many challenges in directing these proteins to their predestined target membrane. The hydrophobic signal peptide or transmembrane domain (TMD) of the nascent protein must be shielded from the aqueous cytosol and its target membrane identified followed by transport and insertion. Components that evolved to deal with each of these challenging steps range from chaperones to receptors, insertases, and sophisticated translocation complexes. One prominent translocation pathway for most proteins is the signal recognition particle (SRP)-dependent pathway which mediates co-translational translocation of proteins across or into the endoplasmic reticulum (ER) membrane. This textbook example of protein insertion is stretched to its limits when faced with secretory or membrane proteins that lack an amino-terminal signal sequence or TMD. Particularly, a large group of so-called tail-anchored (TA) proteins that harbor a single carboxy-terminal TMD require an alternative, post-translational insertion route into the ER membrane. In this review, we summarize the current research in TA protein insertion with a special focus on plants, address challenges, and highlight future research avenues.

## Teaser

Update on different pathways and candidates that facilitate membrane insertion of tail-anchored proteins in eukaryotes with a special emphasis on recent insights in plant research.

## Diversity of membrane proteins

Roughly, one-third of the average eukaryotic proteome comprises integral membrane proteins (IMPs) that act, for example, as channels, transporters, or receptors (Hegde and Keenan, 2011). IMPs are found in all organellar membranes within the cell. They reside in the lipid bilayers of the endomembrane system (endoplasmic reticulum [ER], Golgi, trans Golgi network [TGN], multivesicular body [MVB], vacuole, peroxisomes, and plasma membrane [PM]) as well as in the membranes of the semiautonomous cell organelles of chloroplast and mitochondria. To maintain membrane integrity and cellular function, correct targeting and insertion of newly synthesized IMPs have to be guaranteed. For this purpose, dedicated signal sequences and insertion pathways have evolved.

**ADVANCES**

Shared features of all IMPs are strongly hydrophobic transmembrane domains (TMDs); yet, these vary in their sequence, number, and final topology, and thereby define different types of membrane proteins (Guna and Hegde, 2018). However, all IMPs face three fundamental challenges in their biogenesis:

- Research in the last decade revealed several different targeting routes for TA protein transport and translocation into the ER and organellar membranes of eukaryotes.
- The GET pathway described for TA protein insertion in yeast and mammals is partially conserved in Arabidopsis, where loss of function leads to defects in root hair growth.
- Absence of the coreceptor for TA protein docking and insertion at the ER membrane in the context of the GET pathway in plants phenocopies other *get* lines.
- Sequence information confirms conservation of alternative yeast pathways in plants, while functional data currently remain elusive.
- TA protein import into the ER membrane was mainly studied in yeast and mammalian cell culture, but plants have proven to be ideal models to gain a deeper understanding of these pathways in an organismal context and to study their functional impact on multicellular systems.

1. the nascent protein including its nonpolar TMD(s) must navigate through the aqueous cytosolic environment before reaching the membrane. As exposure of the lipophilic TMDs within the cytosol would lead to premature aggregation, chaperoning proteins are needed which recognize and shield the TMDs until their insertion into the hydrophobic bilayer;
2. IMPs with varying numbers of TMDs and either luminally or cytosolically facing peptide stretches require membrane-bound receptors that aid in the insertion process and guarantee correct orientation within the membrane;
3. finally, targeting sequences (e.g. retention motifs) within the protein need to be recognized to facilitate delivery to the corresponding target membrane (ER and secretory pathway versus organellar membranes; Pedrazzini et al., 1996).

## Of signal recognition and translocons

To cope with the challenges mentioned above, various strategies evolved in eukaryotes were described by scientists in the past decades. Günter Blobel was awarded the 1999 Nobel Prize in Physiology or Medicine “*For the discovery that proteins have intrinsic signals that govern their transport and localization in the cell*” (Celebrating 20 years of cell biology, 2019). Together with David Sabatini, Blobel had postulated the “signal hypothesis” some 30 years earlier (Sabatini and Blobel, 1970). Although a hypothesis at first and rejected by many at the time, it turned out to be correct and found its way into the textbooks. The majority of secretory proteins or IMPs utilize this signal recognition particle (SRP)-dependent pathway and enter the ER through the Sec61 translocon which was later discovered and similarly earned its discoverer Randy Schekman a Nobel prize (Novick et al., 1980; Deshaies et al., 1991) shared with James Rothman and Thomas Südhof “*for their discoveries of machinery regulating vesicle traffic, a major transport system in our cells*” (Wickner, 2013). The pathway is also referred to as “co-translational” as it targets and inserts proteins into the ER during their synthesis (Anderson et al., 1982).

Translocation starts with the extrusion of a nascent polypeptide chain from the ribosome exit channel. SRP recognizes ribosomes with either an N-terminal signal sequence or TMD of a nascent protein (Ogg and Walter, 1995; Shao and Hegde, 2011). Subsequent binding of SRP to the ribosome transiently arrests protein synthesis by blocking further tRNA entry (Lakkaraju et al., 2008; Richter and Coller, 2015). Targeting to the ER membrane of the SRP/ribosome–nascent chain (RNC) complex is induced by the binding to the SRP receptor (SR) in a GTP-dependent manner (Gilmore et al., 1982a, 1982b ). Subsequent conformational changes lead to interaction with the Sec61 translocon, unloading of RNC from SRP to Sec61 and determine the duration of the translational pause. GTP hydrolysis triggers the disassembly of SRP from SR and recycling of the components for additional rounds of protein targeting (Song et al., 2000; Shao and Hegde, 2011).

During co-translational insertion, two mechanisms protect the TMD from the aqueous cytosol:

1. early targeting of the TMD by SRP and maintenance of this connection until docking at the Sec61 channel to ensure minimal exposure to the cytosol before integration and
2. translational slowdown that prevents translation of additional, subsequent TMDs into the cytosol (Walter and Blobel, 1981; Pechmann et al., 2014).

Little is known about an Archaeplastida Sec61 translocon, although such a fundamental mechanism is undoubtedly conserved in plants. Three homologs of each, the central pore Sec61α as well as the two subunits Sec61β and Sec61γ, are encoded in the Arabidopsis (*Arabidopsis thaliana*) genome. While functional data are lacking, physical interaction of *At*Sec61α1 with *At*Sec61β1 and *At*Sec61γ1 was shown by our group (Mehlhorn et al., 2018). In addition, the translocon-associated proteins *At*Sec62 and Sec63 (*At*ErdjA and *At*Erdj2B) are conserved as well (Mitterreiter et al., 2020). Together with the tetratricopeptide repeat protein *At*TPR7, both are probably involved in a chaperone-assisted post-translational import of small peptides in Arabidopsis (Schweiger et al., 2012).

## Tail-anchored proteins

The SRP/Sec61 co-translational pathway reaches its limits, though, when signal sequences or TMDs are lacking within the N-terminal part of the protein. This is in particular the case for type II-orientated membrane proteins that feature a TMD close to their C-terminal end and are referred to as tail-anchored (TA) proteins (Borgese et al., 2003). To distinguish these from other type II proteins, the C-terminal (after translocation: luminal) stretch should by definition be no longer than approximately 30 amino acids (Borgese et al., 2003). This is roughly the length of a peptide stretch within the ribosomal exit channel (Voss et al., 2006). Proteins with such feature are released from the ribosome when their TMD is disclosed to the cytosolic environment. To prevent aggregation of the hydrophobic TMD within the aqueous cytosol, immediate action of chaperones is required aiding in shuttling and post-translational translocation (Pedrazzini, 2009; Johnson et al., 2013).

TA proteins make up to approximately 3%–5% of all IMPs and can be found in almost all cellular membranes (Abell and Mullen, 2011). In Arabidopsis, around 500 TA proteins were predicted in silico (Kriechbaumer et al., 2009). They play key roles in many vital processes such as vesicle trafficking, apoptosis, translocation of other proteins, ubiquitination, signal transduction, enzymatic reactions, or regulation of transcription (Borgese et al., 2003; Kriechbaumer et al., 2009). Some TA proteins even take part in translocation of other membrane proteins as subunits of translocation machineries such as the Sec61β subunit of the SEC61 translocon, or Translocase of outer membrane 22 (Tom22) and Translocase of chloroplast 33 (Toc33) of the mitochondrial and chloroplast import machineries. Additionally, most of the soluble N-ethylmaleimide-sensitive factor attachment receptors (SNAREs) which facilitate vesicle fusion in eukaryotic cells, are TA proteins (Neveu et al., 2020). Their prominent role in many physiological processes is reflected by the dramatic phenotypes associated with their *loss-of-function* lines, ranging from conditional sensitivity toward pathogens to embryonic lethality (Lipka et al., 2007).

## Anchoring in the ER membrane

**Figure 1:**
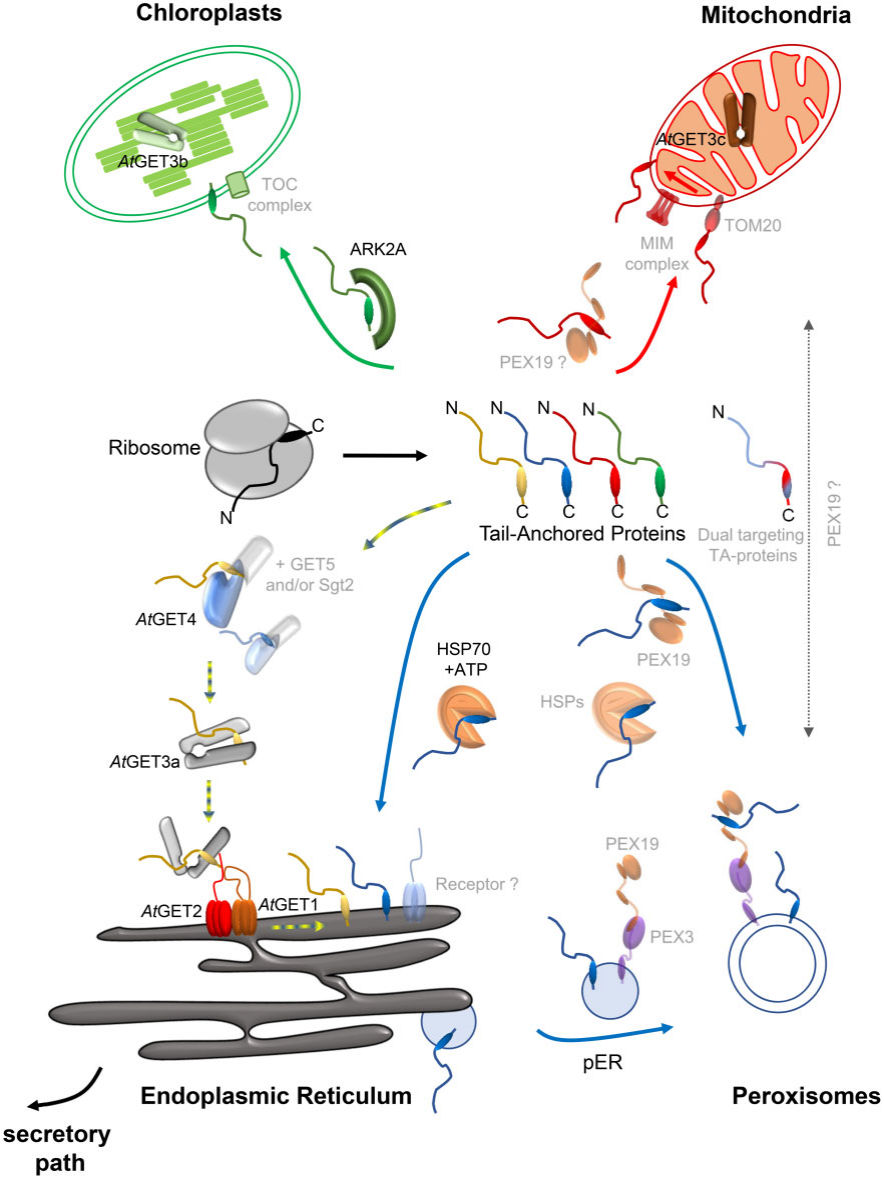
Graphical summary depicting translocation pathways of TA-proteins in plants. Detailed description of the pathways can be found in the text. ER-destined TA proteins (yellow), peroxisomal TA proteins (blue), mitochondrial TA proteins (red), chloroplastidic TA proteins (green), and dual-targeted TA proteins (mitochondria/peroxisomes, dashed arrow, red-blue TA-protein). Opaqueness generally refers to proposed mechanisms/proteins/complexes which may be involved in TA protein translocation in plants but still require experimental validation.

The seemingly textbook example for post-translational membrane insertion of TA proteins into the ER is the Guided Entry of Tail-anchored (GET) proteins pathway (Figure 1) which was initially identified in mammals and yeast (Stefanovic and Hegde, 2007; Schuldiner et al., 2008).

In yeast, nascent TA proteins are recognized immediately after emergence from the ribosomal exit tunnel through a tripartite pretargeting complex consisting of small glutamine-rich tetratricopeptide cochaperone 2 (Sgt2), Get4, and Get5 (Chang et al., 2010; Wang et al., 2010). A functional mammalian homolog of Get4/5 is the B-cell lymphoma 2 (BCL2)-associated athanogene cochaperone 6 (BAG6) complex comprising BAG6, TMD recognition complex 35 (TRC35) and ubiquitin-like domain (UBL)-containing protein 4A (UBL4A), which works in cooperation with small glutamine-rich tetratricopeptide repeat-containing protein α, the mammalian Sgt2 ortholog (Mariappan et al., 2010; Johnson et al., 2013). While Sgt2 alone is ineffective in binding TA proteins, Get4/5 assists this process by bridging and facilitating TA protein transfer from Sgt2 to the cytosolic ATPase Get3 (in mammals TRC40 or Asna1; Suloway et al., 2009; Simpson et al., 2010; Chartron et al., 2011; Gristick et al., 2014). BAG6 triages nascent TA proteins in either an insertion competent fraction or destined for proteasomal degradation (Leznicki and High, 2012; Shao et al., 2017). Recent work has now demonstrated that polyubiquitinated TA proteins can circumvent recognition by BAG6 and still be inserted via the (mammalian) TRC40 pathway and subsequent deubiquitination (Culver and Mariappan, 2021).

Key component of the pathway is the dimeric ATPase Get3. Its subunit interaction is stabilized by a Zn^2+^ ion coordinated by a CxxC motif (Mateja et al., 2009; Simpson et al., 2010). Get3 consists of a nucleotide-binding pocket and a TA protein-binding domain and undergoes conformational changes dependent on its nucleotide-binding state (Wereszczynski and McCammon, 2012). In a nucleotide-free state, Get3 is in an open conformation while binding of ATP leads to a closed dimer, thereby creating a hydrophobic groove that binds and shields the TMD of TA proteins (Mateja et al., 2009; Wereszczynski and McCammon, 2012; Mateja et al., 2015). It was demonstrated that, unlike SRP, Get3 does not associate with ribosomes (Stefanovic and Hegde, 2007). Get3 shuttles the client protein to the ER membrane receptors consisting of a heteromeric complex of Get1 (WRB in mammals; Vilardi et al., 2011; McDowell et al., 2020) and Get2 (CAML in mammals; Yamamoto and Sakisaka, 2012; Vilardi et al., 2014). The long cytosolic N-terminal domain of Get2 mediates the tethering of the Get3–TA protein complex (Mariappan et al., 2011; Wang et al., 2011). Interaction of Get2 only takes place with a nucleotide-bound Get3 which is also compatible with TA protein binding (Denic et al., 2013). Hydrolysis of ATP opens the Get3 dimer. This conformational change disrupts the hydrophobic groove releasing the bound TA protein and providing it for insertion by the Get1–Get2 insertase (Wang et al., 2014; Zalisko et al., 2017). Intriguingly, Get1 and Get2 compete for Get3 binding via overlapping binding sites (Stefer et al., 2011; Denic et al., 2013), although the interaction between Get3 and a coiled-coil domain of Get1 occurs only with an open, nucleotide-free Get3 (Mariappan et al., 2011). Rebinding of ATP returns Get3 into a closed conformation, thereby weakening the Get3–Get1 interaction which leads to dissociation of Get3 from the membrane and recycling for another round of TA protein loading via the pre-targeting complex Sgt2/Get4/Get5 (Stefer et al., 2011; Suloway et al., 2012).

It is noteworthy that TA protein recognition from the ribosome to the membrane is assisted by heat-shock proteins (Rabu et al., 2008; Craig, 2018). Recently, the involvement of J-domain proteins involved in the TA protein handover from Hsp70 to Sgt2 in yeast has been demonstrated (Cho and Shan, 2018; Cho et al., 2021).

## It GET’s complicated in plants

In Arabidopsis, a high degree of conservation was presumed from an in silico search of GET components (Abell and Mullen, 2011; Duncan et al., 2013). Four years later, the existence and function of a plant GET pathway were demonstrated by two groups, independently (Srivastava et al., 2017; Xing et al., 2017) although some of its components still remain elusive. While a functional Get4 ortholog (At5g63220) was identified in plants, its partner proteins within a putative pre-targeting complex could not be determined as too many potential candidates exist. Based on sequence similarities, there are multiple putative Sgt2 and Get5 orthologs in Arabidopsis, the latter features a ubiquitin-like domain which is present in a wide range of proteins (Paul et al., 2013; Srivastava et al., 2017; Xing et al., 2017). BAG6, the protein that bridges the interaction of Ubl4A to TRC35 within a mammalian pre-targeting complex, is lacking in yeast (Leznicki et al., 2013). Interestingly, a putative BAG6 ortholog (Table 1) exists in Arabidopsis and is involved in triggering autophagy in response to pathogen attack (Li et al., 2016); however, at present, an involvement in a plant GET pathway remains elusive.

**Table 1:** Arabidopsis orthologs of yeast and mammalian TA protein insertion pathways

GET pathway				
Yeast/mammalian	AGI Code	Gene name^a^	Protein localization	References
GET1/WRB	At4g16444	AtGET1	ER membrane^b^	Srivastava et al., 2017; Xing et al., 2017
GET2/CAML	At4g32680	AtGET2	ER membrane^b^	Asseck et al., 2021
GET3/TRC40	At1g01910	AtGET3^a^	Cytosol^b^	Srivastava et al., 2017; Xing et al., 2017
	At3g10350	AtGET3^b^	Chloroplast stroma^b^	
	At5g60730	AtGET3^c^	Mitochondria matrix^b^	
GET4/TRC35	At5g63220	AtGET4	Cytosol^b^	Srivastava et al., 2017; Xing et al., 2017
GET5/UBL4A	At1g55060	UBQ12	Cytosol^c^	Srivastava et al., 2017
SGT2/SGTA	At4g08320	TPR8	Nucleus^c^	Srivastava et al., 2017
BAG6	At2g46240	BAG6	Nucleus^c^	PSI-BLAST, TAIR

SND pathway				

SND1	Not found	–	–	PSI-BLAST
SND2	At4g30500At2g23940	AtSND2^a^	Plasma membrane^c^	PSI-BLAST
		AtSND2^b^	ER membrane^c^	
SND3	Not found	–	–	PSI-BLAST

ER Membrane Complex (EMC)				

EMC1	At5g11560	PNET5	ER membrane^c^	PSI-BLAST, TAIR
EMC2	At3g04830	AtEMC2^a^	ER membrane^c^	PSI-BLAST, TAIR
	At5g28220	AtEMC2^b^	Cytosol^c^	
EMC3	At4g12590	AtEMC3	ER membrane^c^	PSI-BLAST, TAIR
EMC4	At5g10780	AtEMC4	ER membrane^c^	PSI-BLAST, TAIR
EMC5	At5g03345	PRCE2	ER membrane^c^	PSI-BLAST, TAIR
EMC6	At5g49540	AtEMC6	Plasma membrane^c^	PSI-BLAST, TAIR
EMC7	At2g25310	AtEMC7^a^	ER membrane^c^	PSI-BLAST, TAIR
	At4g32130	AtEMC7^b^	ER membrane^c^	
EMC8/9	At5g55940	EMB2731	ER membrane^c^	PSI-BLAST, TAIR
EMC10	Not found	–	–	PSI-BLAST

a Found annotated at TAIR (Arabidopsis.org) or—if designated as unknown protein—our suggestion for future use.

b Experimentally validated, see referenced publication for details.

c Predicted using SUBA (suba.live).

PSI, Position-specific iterative

Other than Get1/WRB, Get2/CAML has no sequence ortholog in plants. However, only recently, a functional Get2/CAML homolog has been identified in Arabidopsis using affinity purification-mass spectrometry (Asseck et al., 2021). Despite low sequence similarity, the overall structure comprising three TMDs and a cytosolic N-terminal stretch of basic amino acid residues seem to be evolutionarily conserved to maintain a common function (Asseck et al., 2021). Position-specific iterative- basic local alignment search tool (BLAST) analysis of the human CAML sequence revealed co-selection of the two functional domains, allowing the identification of orthologous genes also in distant phyla (Borgese, 2020; Asseck et al., 2021). In mammals, the two subunits of the GET receptor complex have been shown to depend on each other for expression and are degraded in the absence of the binding partner (Carvalho et al., 2019; Inglis et al., 2020). Similarly, Get1 deficiency in yeast leads to a reduced protein level of Get2 and vice versa, demonstrating reciprocal regulation of these two proteins (Schuldiner et al., 2008; Stefer et al., 2011). In Arabidopsis, however, the relationship between the receptor components seems to be distinct from that in Opisthokonts. In the absence of its co-receptor, *At*GET2 is still expressed but no longer interacts with the targeting factor *At*GET3a (Asseck et al., 2021).

There are additional, intriguing differences among Archaeplastida GET components such as three different GET3 proteins that were identified in Arabidopsis (namely *At*GET3a, *At*GET3b, and *At*GET3c). In silico comparison of these three paralogs revealed two distinct clades (GET3a and GET3bc) present in the Archaeplastida and SAR supergroup but not in Opisthokonts and Amoebozoa, indicating a duplication event in the evolution of eukaryotes (Xing et al., 2017; Farkas et al., 2019). However, orthologs of *At*GET3c seem to be *Brassicaceae*-specific, whereas several copies of *At*GET3b orthologs can exist in other plant species (Bodensohn et al., 2019). Obvious differences between Archaeplastida GET3 proteins and Opisthokont Get3 include:

1. the conserved CxxC motif necessary for the coordination of a zinc ion and dimer formation (see above) is lacking in GET3a but not in the GET3bc clade despite *At*GET3a retaining the ability to form dimers (Xing et al., 2017). Instead, in GET3a an ExxE motif and additional acidic residues adjacent to the site that usually bears the CxxC motif in other species’ sequences may take over metal ion coordination and dimer stabilization (Farkas et al., 2019);
2. an approximately 30 amino acid long, strongly charged extension was only found in the GET3a clade and suggested to be involved in dimerization (Farkas et al., 2019);
3. *At*GET3a is targeted to the cytosol and probably recruited to the ER membrane as it can be found in microsomal fractions (Srivastava et al., 2017; Bodensohn et al., 2019), which might represent the receptor-bound state. *At*GET3b, however, is located within the stroma of chloroplasts and *At*GET3c in the matrix of mitochondria (Xing et al., 2017). Their organellar function is currently not understood (Zhuang et al., 2017; Bodensohn et al., 2019); and
4. while all three orthologs possess the ATPase motif, GET1 and GET4 binding residues are only conserved in *At*GET3a. Consistent with this finding, only *At*GET3a interacts with *At*GET4 and *At*GET1 but neither *At*GET3b nor *At*GET3c (even in truncated, cytosolic forms; Xing et al., 2017). This suggests that only the cytosolic *At*GET3a plays a role in a canonical ER GET pathway in plants.

## GETting knocked out—phenotypic consequences

But there remain more mysteries. So far only two TA proteins have been identified that show reduced membrane insertion in *Atget* mutants, the pollen-specific SNARE protein SYP72 (Srivastava et al., 2017), and the root-hair-specific SNARE protein SYP123 (Xing et al., 2017). The GET pathway is considered as the main route for post-translational TA protein insertion into the ER. Contrary to such an implied vital role, yeast *loss-of-function* strains are viable under normal growth conditions (Schuldiner et al., 2008) and the lethality under oxidative stress likely relates to the function of *Sc*Get3 as a chaperone for unfolding soluble proteins (Voth et al., 2014). Later analysis of yeast TA proteins revealed that only 2 out of 46 potential client proteins show dependency on the presence of an intact GET pathway. Nonetheless, knockout of the mammalian ortholog TRC40 leads to embryo lethality in mice (Mukhopadhyay et al., 2006) and severe organ defects in induced *get* mutants (Lin et al., 2016; NorLin et al., 2016; Vogl et al., 2016). One could conclude from this that among multicellular Opisthokonts, an intact GET pathway became indispensable for survival.

The data in other multicellular organisms such as plants, however, rule out such a general conclusion. In Arabidopsis, loss of GET pathway function clearly causes effects such as increased ER-stress levels (Srivastava et al., 2017) and reduced root hair length (Xing et al., 2017), yet no pleiotropic phenotypes, let alone seedling or embryo lethality, was observed. Such strong phenotypes, however, should be expected considering that certain vital TA proteins such as the cytokinesis-specific SNARE KNOLLE (Lauber et al., 1997) do not reach their target membrane.

With the implication that the GET pathway is the major hub for TA protein insertion in the ER, the question is justified whether this can hold true with respect to such mild phenotypes and whether or not backup systems have evolved. An alternative explanation would be that a plant GET pathway evolved additional/alternative function(s) instead/apart from TA protein insertion. The latter suggestion is supported by an immunoprecipitation mass spectrometry (IP-MS) analysis where only 23 TA proteins interacted with *At*GET3a-GFP (Xing et al., 2017) which is <5% of all predicted TA proteins in Arabidopsis (Kriechbaumer et al., 2009). Thus, it seems that in plants, the GET pathway might not play a—not to mention *the*—major role in TA protein insertion; or at least that plants have evolved alternative mechanisms to secure TA protein insertion in case one route breaks down.

## GET alternatives

The dispensability of Arabidopsis GET components for general plant growth and survival with merely an effect on root hair growth (Xing et al., 2017; Asseck et al., 2021) allows speculation regarding the existence of a yet-undiscovered alternative insertion pathways in plants that might redundantly substitute TA protein insertion into the ER membrane.

In a pioneering effort, an SRP-independent targeting (SND) pathway consisting of three genetically linked proteins localizing to the cytosol (Snd1) or ER membrane (Snd2 and Snd3) was identified in yeast (Aviram et al., 2016). Here, cytosolic Snd1 is predicted to interact with ribosomes, co-translationally capturing nascent proteins, whereas Snd2 and Snd3 associate with the Sec61 translocon acting as putative receptors. The SND pathway was initially described as a pathway for IMPs harboring an internal TMD, and its loss leads to mislocalisation of these proteins. It was shown that all three Snd proteins act in the same pathway and it additionally serves as a safeguard for both SRP-dependent insertion and the GET pathway. As for the get knockouts, *SND* deletion did not affect the viability of *Saccharomyces* under normal growth conditions. Interestingly, double knockouts between *SND* and *GET* are nonviable, suggesting a compensatory role of TA protein delivery to the ER (Aviram et al., 2016).

In mammals, homologs of Snd1 and Snd3 have not been found, yet an Snd2 homolog (TMEM208 or hSND2) was identified and its localization to the ER confirmed (Hassdenteufel et al., 2017). In two independent studies, the function of hSND2 in TA protein biogenesis shown as deletion leads to decreased TA protein insertion (Casson et al., 2017; Hassdenteufel et al., 2017). Interestingly, loss of hSND2 is compensated by upregulation of the SRP receptor SRα, which was shown to aid in an SRP-dependent post-translational insertion of some client TA proteins (Casson et al., 2017; Hassdenteufel et al., 2017). In Arabidopsis, two sequence paralogs for Snd2 can be identified via BLAST search, but no obvious orthologs for Snd1 or Snd3 (Table 1). It remains to be seen whether an SND pathway is functionally conserved and which proteins pair up with SND2 in plants to facilitate such function.

Another recently discovered post-translational insertase for ER-destined TA proteins with TMDs of moderate-to-low hydrophobicity is the ER membrane complex (EMC). In semi-permeabilized cells silenced for EMC components, integration of the mammalian ER-resident enzyme squalene synthase and four other TA proteins with similar hydrophobic TMD characteristics failed. Calmodulin seems to play a role as a chaperone in this pathway (Guna et al., 2018; Volkmar et al., 2019).

Putative orthologs for all 9–10 components of the mammalian EMC can be found in plants through sequence homology (Table 1). Whether a similar function is associated with these proteins in Arabidopsis or which other proteins are involved within a putative plant EMC complex is currently unresolved. It is noteworthy, that EMC3 as well as Get1 are ER-resident homologs of the Oxa1/Alb3/YidC family of insertases that facilitate co- and post-translational insertion of transmembrane proteins into the inner mitochondrial membrane (Oxa1), the thylakoid membrane (Alb3), and the inner membrane of bacteria (YidC), respectively (Anghel et al., 2017; Samuelson et al., 2000).

The SEC61 translocon and its auxiliary proteins SEC62/SEC63 make use of heat-shock proteins to provide an additional post-translation pathway (Abell et al., 2007; Wu et al., 2019). In Arabidopsis, *At*TPR7 together with the translocon-associated proteins *At*Sec62 and *At*Erdj2 (*At*SEC63) seems to facilitate heat-shock protein-mediated delivery of proteins for post-translational translocation (Schweiger et al., 2012; Schweiger and Schwenkert, 2013). Loss of *At*Sec62 impairs plant growth and reduces male fertility (Mitterreiter et al., 2020), yet it remains to be dissected whether the causative effect of this phenotype is an impairment in translocation or an interference in ER-phagy (FumagalLi et al., 2016; Hu et al., 2020).

## Insertion of TA proteins in other organelles

Translocation to the ER may be the major route for most TA proteins, yet post-translational insertion requires recognition of the target membrane ahead of distribution. This is even more challenging for plants with one additional endomembrane compared with other eukaryotic cells. To distinguish between different destination membranes, targeting information is required within the TA protein.

More than two decades of research in TA proteins has unveiled properties and motives that seem important for endomembrane distinction; however, many candidates still seem to be exempt from rules (Borgese et al., 2001, 2019). These rules comprise targeting signals encoded in the hydrophobicity of the TMD as well as charge and length of the adjacent C-terminal element (CTE; Beilharz et al., 2003; Borgese et al., 2007; Abell and Mullen, 2011; Marty et al., 2014; Rao et al., 2016; Costello et al., 2017).

For ER targeting, the consensus motif seems to be a long and hydrophobic TMD followed by nonpolar, negative, or no residues in the CTE (Rao et al., 2016).

It is currently proposed that TA proteins of the outer mitochondrial membrane (OMM) show less hydrophobic and shorter TMDs with reduced helical content compared to TA proteins destined to the ER or secretory pathway (Kriechbaumer et al., 2009; Lee et al., 2014; Rao et al., 2016; Chio et al., 2017).

Targeting of some mitochondrial TA proteins to the OMM is also conducted by a moderately positively charged CTE (Marty et al., 2014; Rao et al., 2016). For Fis1, it could be demonstrated that a minimum of four basic residues are needed for mitochondrial localization while mutation of the basic residues in the CTE of some OMM TA proteins changes their destination (Rao et al., 2016). For example, mammalian ER-localized cytb5 with a negatively charged CTE localizes to the OMM when artificially reverted to a positive net charge. This same construct expressed in plant cells, however, is directed to the chloroplast highlighting the challenges associated with the discrimination of multiple destination membranes (Maggio et al., 2007). It was also demonstrated that two cytochrome b5 (cytb5) isoforms—both with positive net charges in their CTE, but a number of putative phosphorylation sites—localize to either the ER or the chloroplast outer envelope (Maggio et al., 2007), which leads to the speculation of phosphorylation as a cue to aid in discriminating target membranes through reversion of a positive net charge. Mitochondrial targeting is also dependent on the distance between TMD and CTE (Marty et al., 2014). Another potent indicator of plant OMM TA proteins is found in the dibasic motif adjacent to the C-terminal part of the TMD (Marty et al., 2014).

In mammals and yeast, no unambiguous amino acid motif for TA protein targeting had been found so far. A recent study in Arabidopsis, however, showed that some plastid outer envelope membrane (OEM) TA proteins harbor a CTE with an RK/ST sequence motif. OEP7.2, which localizes to the OEM, was used for swapping experiments with CTEs of other TA proteins with and without this motif. Only CTE with RK/ST motifs was functionally interchangeable. Thus, they concluded that for a subset of OEM TA proteins, there is a conserved element for plastid targeting (Moog, 2019; Teresinski et al., 2019).

Overall, it seems that the length and hydrophobicity of the TMD with a combination of charge dictates the localization of TA proteins within the cell, while plant OEM TA proteins with a specific motif might be more of an exception.

However, dually targeted TA proteins such as *At*PMD1 to mitochondria and peroxisomes (Aung and Hu, 2011), *At*PAP2 to chloroplast and mitochondria (Sun et al., 2012), or proteins which display multiple targeting [chloroplast, mitochondria, and peroxisomes] as *At*FIS1A (Ruberti et al., 2014), highlight that topogenic information (alone) cannot suffice to discern targeting routes. Nonetheless, the specificity of targeting motifs is interlinked with the binding properties of different chaperones that shepherd their substrate to their destination membrane.

Potentially as a consequence of ambiguous signals, mistargeting occurs against which fail-safe mechanisms evolved: in yeast, the AAA-ATPase Msp1 (Okreglak and Walter, 2014; Wang et al., 2020) recognizes TA proteins wrongly delivered to the OMM and either hands them over for proteasome-mediated degradation or extracts them for correct rerouting (thoroughly reviewed in Wang and Walter, 2020). While such dislocase function also exists in animals (ATAD1, Chen et al., 2014a) a similar function has not been found in plants where a large number of AAA-ATPases exist (Ogura and Wilkinson, 2001).

### Insertion into chloroplasts

The translocation mechanism of TA proteins into the OEM of chloroplasts is currently not well understood. Unassisted insertion dependent on the lipid composition of the membrane and the TA protein CTE has been observed (Qbadou et al., 2003; Pedrazzini, 2009; Dhanoa et al., 2010). Additionally, a cytosolic OEM chaperone, ankyrin repeat-containing protein (AKR2a) was found to play a role for the targeting of some TA proteins to chloroplasts and the delivery of dual-targeted ascorbate peroxidase (APX3) to peroxisomes (Bae et al., 2008; Shen et al., 2010). This observation would argue against its role as a specific chloroplast TA protein insertion factor indicating AKR2a as a rather unspecific chaperone.

Recently, another putative chaperone was detected in the green algae *Chlamydomonas reinhardtii*. Here, an arsenite transporter (*Cr*ArsA1) binds Toc34 and delivers it to chloroplasts (Maestre-Reyna et al., 2017). Intriguingly, two ArsA paralogous genes can be found in the *C. reinhardtii* genome, *Cr*ArsA1 and *Cr*ArsA2. Both are homologs of the cytosolic targeting factors, TRC40 and Get3 (Formighieri et al., 2013). *Cr*ArsA1 and *Cr*ArsA2 have a discrete ligand preference, with *Cr*ArsA1 supposedly carrying TA proteins to the OEM and *Cr*ArsA2 to the ER (Maestre-Reyna et al., 2017). The subcellular localization of ArsA1 homologs in chlorophytes is a matter of debate. While Formighieri et al. (2013) propose *Cr*ArsA1 to be cytoplasmic, its protein sequence clearly features an organellar transit peptide at the N-terminus (Xing et al., 2017; Farkas et al., 2019). Its sequence also suggests a high similarity to other GET3bc clade homologs of Archaeplastida, which are also organellar localized (Xing et al., 2017; Lin et al., 2019). In addition, a recent affinity purification mass spectrometry of the chloroplastic ribosome interactome of *Chlamydomonas reinhardtii* revealed *Cr*ArsA1 lending further support to its stroma rather than cytosolic localization (Westrich et al., 2021).

The Arabidopsis homolog of *Cr*ArsA1 is *At*GET3b, which also features an N-terminal transit peptide and localizes to the stroma of chloroplasts (Xing et al., 2017). However, localizing within the stroma precludes a possible involvement in TA protein insertion at the OEM. One could speculate that AtGET3b is involved in TA protein targeting the inner envelope membrane or thylakoids (Anderson et al., 2019; Bodensohn et al., 2019). While *At*GET3b does not bind to ER-resident *At*GET1 (Xing et al., 2017), interaction assays should first elucidate whether *At*GET3b could potentially bind to the Get1 ortholog Alb3 (At2g28800) or Alb4 (At1g24490), which facilitates membrane protein biogenesis in endosymbiontic organelles (Anghel et al., 2017; McDowell et al., 2021).

### Insertion into mitochondria

Mitochondria have a small semi-autonomous genome, although most of the mitochondrial proteins are encoded by the nuclear genome, synthesized by cytosolic ribosomes, and transported post-translationally into the mitochondria (Neupert, 1997; Pfanner and Geissler, 2001). There are many mitochondrial TA proteins, yet the pathway(s) responsible for their insertion are not clear. It had been reported that insertion of mitochondrial TA proteins depended on the unique lipid composition of the OMM, especially the ergosterol levels (Setoguchi et al., 2006; Kemper et al., 2008; Krumpe et al., 2012) and with the help of peroxisome import factor Pex19 (Cichocki et al., 2018). Moreover, translocation of TA proteins was moderately affected with hampered mitochondrial import complex (MIM) or Tom20 receptors (Thornton et al., 2010; Doan et al., 2020). It is conceivable that Tom20 acts as a receptor, while the MIM complex mediates insertion (Drwesh and Rapaport, 2020). Also, N-terminally GFP-labeled OMM protein Mcp3 mislocalizes to the ER in wild-type yeast but not in *get* knockout strains (Vitali et al., 2018). Apparently, when the mitochondrial import is compromised, TA proteins intended for the OMM are mistargeted to the ER membrane by the GET pathway. This implies that in yeast insertion pathways may compete for client delivery.

*At*Get3c, a homolog of Get3 is found in the mitochondrial matrix of Arabidopsis. Whether or not it is involved in TA protein insertion into the inner membrane of mitochondria is currently unknown. However, its *loss-of-function* line seems to show no obvious growth or cellular defects (Xing et al., 2017). It was speculated that the GET3c variants are *Brassicaceae*-specific, while some GET3b homologs (that localize to chloroplasts in Arabidopsis) were mitochondria localized in the *Fabidae* (Bodensohn et al., 2019). Similar to chloroplasts, a Get1 ortholog is present in the mitochondrial inner membrane (Oxa1). As discussed above, the GET3bc clade lacks the GET1 binding motif (Anghel et al., 2017; Farkas et al., 2019) and has not been demonstrated to interact with or depend on Oxa1 so far.

### Insertion into peroxisomes

Peroxisomes are single membrane, multifunctional organelles with essential roles in development such as scavenging of reactive oxygen species or peroxides, photorespiration, glycolate cycle, and fatty acid β-oxidation (Aung and Hu, 2011; Kao et al., 2018). In contrast to chloroplasts and mitochondria, they neither contain DNA nor possess protein-synthesizing machinery. Peroxisomes are discussed to be ER-derived and early acting peroxin (PEX) proteins such as PEX3, PEX16, and PEX19 help in the peroxisomal genesis but also a division by fission is possible (Kao et al., 2018). Therefore, the acquisition of protein delivery machineries is of great importance for peroxisomal identity.

In mammals and yeast, it was shown that peroxisomal-targeted TA proteins can take two distinct routes, (1) directly from the cytosol or (2) via the ER (Borgese et al., 2019). Both ways depend on the peroxisomal import proteins Pex19 and Pex3. Cytosolic Pex19 binds nascent peroxisomal TA proteins within a hydrophobic groove, thereby stabilizing them. Recognition occurs via the TMD and basic CTE of the TA proteins (Halbach et al., 2006; Yagita et al., 2013; Chen et al., 2014b). The binding of its membrane receptor Pex3 leads to direct insertion into the membrane (Cichocki et al., 2018).

ER-dependent insertion is partially carried out by the GET machinery. For instance, yeast Pex15 is ER-inserted via the GET pathway (Schuldiner et al., 2008; van der Zand et al., 2010). Here, a specialized subdomain within the ER is formed, the so-called peroxisomal ER (pER). Localized budding of peroxisomal vesicles carrying TA proteins and subsequent fusion to existing peroxisomes requires Pex3, Pex19, ATP, and additional yet unidentified cytosolic factors (van der Zand et al., 2010; Lam et al., 2011). Studies on these events proposed a dual functionality of Pex3. Its luminal sequence harbors a sorting signal for delivering Pex3 to the pER, whereas the TMD of Pex3 is important for later directing of the vesicles to peroxisomes (Tam et al., 2005; Fakieh et al., 2013; Chio et al., 2017).

In plants, the peroxisomal-targeted TA protein APX was shown to insert post-translationally dependent on ATP, Hsp70, and an additional, unknown receptor via pER (Mullen and Trelease, 2000). Unassisted insertion can also be observed for some peroxisomal TA proteins as MDAR4 (Lisenbee et al., 2005; Abell and Mullen, 2011). A conserved mechanism for translocation of plant TA proteins as seen in *Opisthokonts* is conceivable; however, exact information is lacking (Cross et al., 2016).

## Future perspectives

The most puzzling discovery in TA protein insertion in plants is certainly with a rather mild phenotype associated with GET *loss-of-function* lines (see Advances). How can this be reconciled with the notion that the GET pathway is universally conserved and acts as *the* textbook pathway for TA protein insertion into the ER? A nonlethal phenotype of a plant that lacks a general membrane insertion pathway of an important subclass of membrane proteins would surely lead to more pleiotropic growth defects. Failure to insert TA proteins—among them the trafficking facilitating SNARE proteins which are required for polar growth and cytokinesis—should lead to embryo lethality “at best”, or developmental arrests in earlier stages such as compromised pollen tube growth. Their absence suggests one or more backup system(s) in place. Existence, identity, and conservation of such systems (e.g. SND, EMC, Table 1) are a major avenue for future research as well as the identification of further GET pathway substrates which may also aid in understanding additional function(s) of a plant GET pathway.

Another obvious question is the precise targeting and distinction of TA proteins to their various destination membranes. A complex combination of physicochemical properties or as in the case of some plant OEM TA proteins, a specific motif (Teresinski et al., 2019) might be the answer. Yet, how exactly dual-targeted TA proteins are sorted is still not clear and a simple solution is unlikely.

A puzzling observation is the additional GET3 paralogs in Archaeplastida (Xing et al., 2017; Farkas et al., 2019). While clade *a* GET3 appears to be functionally related to yeast Get3 and mammalian TRC40, the roles of clade *bc* GET3 remain elusive. All plants likely possess at least one copy of a chloroplast GET3b which might be involved in TA protein targeting to the inner envelope or thylakoids. However, the mitochondrial GET3c seems absent in most plant species, which begs questions about its functional role and evolution (Bodensohn et al., 2019).

These are just some points that require addressing in future research and there is a lot to learn in terms of TA protein insertion in plants (see Outstanding Questions). Other fundamental homeostatic pathways such as cytokinesis (Jurgens, 2005) have significantly diverged among Opisthokonts and Archaeplastida—an evolutionary divide of more than 1.5 billion years—and validated the importance of research into different model species. Nonetheless, evidence for functional conservation of important fundamental processes such as membrane protein insertion remains limited in plants. The vast amount of data gained from research in single-celled models such as bacteria, yeast, and cell culture should be used to inform hypothesis-driven research in plants. Especially, the model plant Arabidopsis and the palette of modern genomic tools established therein will allow a more organismal-focused, phenotypic analyses of these pathways in the context of a multicellular organism.

**OUTSTANDING QUESTIONS**

- Which additional pathways for TA protein insertion exist in plants?
- What alternative functions have evolved for the GET pathway components in Arabidopsis or more generally in plants?
- Why did Archaeplastida evolve organellar variants of the GET3 ATPase and what is (are) their function(s)?
- Is a post-translational pretargeting complex conserved in archaeplastida?

## Notes

D.G.M. and C.G. wrote the manuscript with input from L.Y.A.

The author responsible for distribution of materials integral to the findings presented in this article in accordance with the policy described in the Instructions for Authors (https://academic.oup.com/plphys/pages/general-instructions) is: Christopher Grefen (christopher.grefen@rub.de).
